# Power-Law Dynamics of Membrane Conductances Increase Spiking Diversity in a Hodgkin-Huxley Model

**DOI:** 10.1371/journal.pcbi.1004776

**Published:** 2016-03-03

**Authors:** Wondimu Teka, David Stockton, Fidel Santamaria

**Affiliations:** 1 UTSA Neurosciences Institute, The University of Texas at San Antonio, San Antonio, Texas, United States of America; 2 Biomedical Engineering Program, The University of Texas at San Antonio, San Antonio, Texas, United States of America; The Krasnow Institute for Advanced Studies, UNITED STATES

## Abstract

We studied the effects of non-Markovian power-law voltage dependent conductances on the generation of action potentials and spiking patterns in a Hodgkin-Huxley model. To implement slow-adapting power-law dynamics of the gating variables of the potassium, n, and sodium, m and h, conductances we used fractional derivatives of order *η*≤1. The fractional derivatives were used to solve the kinetic equations of each gate. We systematically classified the properties of each gate as a function of *η*. We then tested if the full model could generate action potentials with the different power-law behaving gates. Finally, we studied the patterns of action potential that emerged in each case. Our results show the model produces a wide range of action potential shapes and spiking patterns in response to constant current stimulation as a function of *η*. In comparison with the classical model, the action potential shapes for power-law behaving potassium conductance (n gate) showed a longer peak and shallow hyperpolarization; for power-law activation of the sodium conductance (m gate), the action potentials had a sharp rise time; and for power-law inactivation of the sodium conductance (h gate) the spikes had wider peak that for low values of *η* replicated pituitary- and cardiac-type action potentials. With all physiological parameters fixed a wide range of spiking patterns emerged as a function of the value of the constant input current and *η*, such as square wave bursting, mixed mode oscillations, and pseudo-plateau potentials. Our analyses show that the intrinsic memory trace of the fractional derivative provides a negative feedback mechanism between the voltage trace and the activity of the power-law behaving gate variable. As a consequence, power-law behaving conductances result in an increase in the number of spiking patterns a neuron can generate and, we propose, expand the computational capacity of the neuron.

## Author Summary

There is increasing evidence that the activity of individual membrane ion channels, conductances, and the firing rate of neurons are history dependent. In this work we studied how history dependent activation of membrane conductances affect the action potential activity of the Hodgkin-Huxley model, a widely used model of action potential generation. In order to implement history dependent activation, we made use of fractional order differential equations. This type of history dependent differential equations are increasingly being used in biomedical sciences to simulate complex phenomena. We use fractional order derivatives to model the kinetic dynamics of the gate variables for the potassium and sodium conductances of the Hodgkin-Huxley model. Our results show that power-law dynamics of the different gate variables result in a wide range of action potential shapes and spiking patterns, even in the case where the model was stimulated with constant current. As a consequence, power-law behaving conductances result in an increase in the number of spiking patterns a neuron can generate and, we propose, expand the computational capacity of the neuron.

## Introduction

The large majority of conductance based neuronal models assume that the membrane voltage and conductances follow a Markov process [1, 2]. As such, the value of each of these variables in the next time point is dependent exclusively on its present state [3]. Increasing evidence shows that this assumption is not applicable all the time. The distribution of closed states of single channels [4, 5], the recovery time from inactivation of individual conductances [6, 7], and the spiking patterns generated over prolonged periods of time [8] show history dependence. If a conductance’s response to a voltage clamp command follows Markov dynamics then the time adaptation of the conductance is described with an exponential function. In contrast, if the adaptation of the conductance is history dependent then its response is usually described with a power-law. The power-law response could be due to the cumulative effect of multiple exponential processes with time constants distributed over a wide range of scales [9]. However, power-laws also arise when the fundamental Markovian assumptions break down with no single time constant describing the behavior of the system and possibly reflecting strong, allosteric, interactions among internal states of the channels [10]. Under such conditions the transitions between states depend on the history of the activity of the channel. While many of the studies on power-law dynamics in single neurons have centered on action potential rates [11–13] and membrane voltage [14–17] little is known of how a power-law behaving conductance could affect the spike generation properties of a neuron.

The natural mathematical tool to implement history dependent power-law dynamics is the fractional order differential equation [18]. For processes that show slow adaptation the order of the fractional derivative (*η*) is less than 1. The value of the fractional order corresponds to the power-law exponent of the process being modeled. We recently introduced the fractional leaky integrate-and-fire model (LIF) [14], which we have used to replicate the firing rate activity of adapting cortical neurons. We have also developed tools to efficiently integrate such equations [19]. Other groups have used the fractional derivative of the voltage to study the Hodgkin-Huxley [15–17] model or to model the power-law firing rate adaptation observed in cortical and brain stem neurons [12, 13]. Fractional order dynamics is being increasingly used through computational biology sub-disciplines to model complex systems that show history dependence and power-law dynamics [20].

Here we study the effects of power-law behaving conductances in a biophysical model of spiking activity, the Hodgkin-Huxley model. We systematically modified the dynamics of the gating variables of the potassium (n) and sodium (m and h) conductances to generate power-law history dependent activity. Our results show the emergence of a wide range of spiking behaviors in response to constant stimulation as a function of the fractional order in the different activation/inactivation variables. In the case of the n gate, the neuron shows reduction of spiking response and emergence of sub-threshold oscillations. While power-law behavior in the h gate results in bursting activity and pseudo-plateau potentials. This emergent richness in spiking activity, while only modeling two conductances, allows to study the effects of power-law behavior in neuronal activity. Computationally, we suggest that power-law conductance behavior allows neurons to increase their coding capacity.

### Methods

The Hodgkin and Huxley model is [1]

$$C\frac{dV}{dt}=-\left(g_{m}\left(V-E_{l}\right)+\bar{g_{K}}n^{4}\left(V-E_{K}\right)+\bar{g_{Na}}m^{3}h\left(V-E_{Na}\right)\right)+I$$ (1)

where C is the membrane capacitance; V is the membrane voltage; g_m_ is the passive conductance; E_l_ is the leak reversal potential; $\bar{g_{K}}$ and $\bar{g_{Na}}$ are the maximum potassium and sodium conductances, respectively; E_K_ and E_Na_ are their reversal potentials, and I is the input current. The gating variables n, m, and h are defined by the general equation

$$\frac{dx}{dt}=\alpha_{x}\left(V\right)\left(1-x\right)-\beta_{x}\left(V\right)x$$ (2)

where x = [n, m, h], the function *α* is the forward rate, and *β* is the backward rate. The gating variables n and m are known as activation variables while h is an inactivation variable. The functional forms of n, m, and h are [1]:

$$\alpha_{n}\left(V\right)=\frac{0.1-0.01\left(V-V_{0}\right)}{e^{1-0.1\left(V+V_{0}\right))}-1}$$ (3)

$$\beta_{n}\left(V\right)=0.125e^{-\left(V-V_{0}\right)/80}$$ (4)

$$\alpha_{m}\left(V\right)=\frac{2.5-0.1\left(V-V_{0}\right)}{e^{2.5-0.1\left(V-V_{0}\right))}-1}$$ (5)

$$\beta_{m}\left(V\right)=4e^{-\left(V-V_{0}\right)/18}$$ (6)

$$\alpha_{h}\left(V\right)=0.07e^{-\left(V-V_{0}\right)/20}$$ (7)

$$\beta_{h}\left(V\right)=\frac{1}{1+e^{\left(3-0.1\left(V-V_{0}\right)\right))}}$$ (8)

In this work we systematically study the effects on the spiking activity of the Hodgkin-Huxley model to the implementation of fractional dynamics on each of the gating variables:

$$\frac{d^{\eta }x}{dt^{\eta }}=\alpha_{x}\left(V\right)\left(1-x\right)-\beta_{x}\left(V\right)x$$ (9)

where we use the Caputo definition [21] of the fractional derivative for η<1

$$\frac{d^{\eta }f}{dt^{\eta }}=\frac{1}{\Gamma \left(1-\eta \right)}\int_{0}^{t}\frac{f^{\prime }\left(t\right)}{{\left(t-u\right)}^{\eta }}du$$ (10)

where Γ is the Gamma function. The fractional derivative value is the result of integrating the activity of the function over all past activities weighted by a function that follows a power-law. The weighted past values are called the memory trace. As opposed to the first derivative, the fractional derivative provides information over all past activity. We numerically integrate the fractional derivative using the L1 scheme [22],

$$\frac{d^{\eta }x\left(t_{N}\right)}{dt^{\eta }}\approx \frac{{\left(dt\right)}^{-\eta }}{\Gamma \left(2-\eta \right)}\left[\sum_{k=0}^{N-1}\left[x\left(t_{k+1}\right)-x\left(t_{k}\right)\right]\left[{\left(N-k\right)}^{1-\eta }-{\left(N-1-k\right)}^{1-\eta }\right]\right]$$ (11)

where 0<η≤1, t_k_ = k dt, N = t_N_/dt, and dt = 0.001 ms. By combining this equation and the gating dynamic equation and solving for x at time t_N_ we obtain the equation that we use to integrate the function

$$x\left(t_{N}\right)\approx dt^{\eta }\Gamma \left(2-\eta \right)\left[\alpha_{x}\left(V,t_{N-1}\right)\left(1-x\left(t_{N-1}\right)\right)-\beta_{x}\left(V,t_{N-1}\right)x\left(t_{N-1}\right)\right]+x\left(t_{N-1}\right)-\left[\sum_{k=0}^{N-2}\left[x\left(t_{k+1}\right)-x\left(t_{k}\right)\right]\left[{\left(N-k\right)}^{1-\eta }-{\left(N-1-k\right)}^{1-\eta }\right]\right]$$ (12)

Where, again, x = [n, m, h]. The first two components of the right hand side of the equation are the solution of the classical differential equation. The last component of the equation is the memory trace. We have recently developed efficient ways to computationally solve these equations [19]. The memory trace is the last part of Eq 12

$$-\left[\sum_{k=0}^{N-2}\left[x\left(t_{k+1}\right)-x\left(t_{k}\right)\right]\left[{\left(N-k\right)}^{1-\eta }-{\left(N-1-k\right)}^{1-\eta }\right]\right]$$ (13)

The large number of simulations performed for this study were managed using our recently developed simulator workflow manager (NeuroManager) [23]. In brief, NeuroManager is an object-oriented application written in MATLAB (Natick, MA) that automates the workflow of submitting neuroscience simulations. The simulations in this paper were run by NeuroManager using a heterogeneous set of resources ranging from local UNIX servers (multi-core XEON processors), institutional clusters (Cheetah cluster at the UTSA Computational Biology Initiative, www.cbi.utsa.edu), and national resources (Stampede Cluster at the Texas Advanced Computing Center, www.tacc.utexas.edu). NeuroManager allows the user to isolate the free parameters of the simulations and define them as an Input Parameter Vector and organizes the results and products of each simulation. All code is available at GitHub (https://github.com/SantamariaLab/PowerLawHH), and the ModelDB database (https://senselab.med.yale.edu/ModelDB accession number 187600).Unless otherwise indicated the simulations use the following parameter values assuming 1 cm^2^ of membrane: C = 1 μF, $\bar{g_{Na}}$ = 120 mS, $\bar{g_{k}}$ = 36 mS, $\bar{g_{m}}$ = 0.3 mS, E_Na_ = 50 mV, E_K_ = -77 mV, and E_L_ = -54 mV. For all the simulations we used the same initial conditions: m = 0.0529; h = 0.5960; n = 0.3177; and V_0_ = -65 mV, which produced a zero change in voltage in the classic case. To calculate the value of the Mittag-Leffler function (see below) we used the algorithm developed by I. Podlubny and M. Kacenak (www.mathworks.com/matlabcentral/fileexchange/8738-mittag-leffler-function).The value of the power-law behaving gate was calculated using Eq 12 and the value of all other variables in Eq 1 were calculated using a Runge-Kutta method of 4^th^ order.

## Results

Our goal was to determine the effects of power-law activation of membrane conductances on spiking activity. The natural mathematical way to implement power-law dynamics is by using fractional order differential equations. We modified a Hodgkin-Huxley model to incorporate fractional order gating variables. First, we provide a theoretical justification of the model and then describe the effects of having fractional order dynamics on the individual gates. The analyses of the gate variables provide a method to determine whether an experimentally measured conductance is following a power-law process.

### Theoretical justification

Traditionally, a single ion channel is described to have an open and closed states. The closed state can be composed of multiple ‘hidden’ states. Under Markovian assumptions states are independent and their residence times follow exponential dynamics. To produce power-law dynamics of the open-close transitions one can assume the existence of a large number of hidden states. Under such a model the state of the channel can be described as a diffusion process over a large number of traps. These types of models are well known to produce anomalous diffusion, a power-law behavior [24] and have been shown to replicate single channel dynamics [25]. It is also possible that the residence times do not follow exponential dynamics, due to internal state interactions or temporal correlations [25].

A purely power-law process does not have a mean residence time [26]. This would result in the absence of a stationary response. Since it is possible to get stationary responses when measuring conductance dynamics, it is necessary to assume that a channel can have a normal and power-law transitions. As such, we develop our model by expanding the Hodgkin-Huxley gating dynamics (Eq 2) to have both classical and power-law components

$$\frac{dx}{dt}=r_{0}\left[\alpha_{x}\left(V\right)\left(1-x\right)-\beta_{x}\left(V\right)x\right]+\sum_{i=1}^{m}r_{i}\frac{d^{1-\eta_{i}}}{dt^{1-\eta_{i}}}\left[\alpha_{x}\left(V\right)\left(1-x\right)-\beta_{x}\left(V\right)x\right]$$ (14)

The sum on the right hand side of the equation describes multiple gating processes with different fractional order dynamics that describe memory dependent activity. We chose to use the same reaction rates (*α*_*x*_(*V*) and *β*_*x*_(*V*)) for simplicity and then scale them with the factors *r*_*i*_, *i* = 0 *to m*. This is similar to the fractional relaxation equation of [26]. This full model describes a system that has a finite mean residence time (classical component) with the perturbation from power-law processes. We can write the same equation in a compact form

$$\frac{dx}{dt}=\sum_{i=0}^{m}r_{i}\frac{d^{1-\eta_{i}}}{dt^{1-\eta_{i}}}\left[\alpha_{x}\left(V\right)\left(1-x\right)-\beta_{x}\left(V\right)x\right]$$ (15)

We define *η*_0_ = 1 and *r*_0_ = 1 so in the case when *m* = 0 the model reduces to Eq 2. For *m* = 1 the system models a mixture of the classical and a single fractional order process. In our case, we assume that the rate of transition of the classical model is much smaller than the rate of the fractional model (*r*_0_ ≪ *r*_1_). This means that the fractional dynamics occur much faster than the classical process. Thus we can approximate the dynamics as (*r*_1_→1)

$$\frac{dx}{dt}\approx \frac{d^{1-\eta }}{dt^{1-\eta }}\left[\alpha_{x}\left(V\right)\left(1-x\right)-\beta_{x}\left(V\right)x\right]$$ (16)

Re-arranging the fractional order operator yields our model

$$\frac{d^{-1+\eta }}{dt^{-1+\eta }}\frac{dx}{dt}=\left[\alpha_{x}\left(V\right)\left(1-x\right)-\beta_{x}\left(V\right)x\right]$$ (17)

$$\frac{d^{\eta }x}{dt^{\eta }}=\left[\alpha_{x}\left(V\right)\left(1-x\right)-\beta_{x}\left(V\right)x\right]$$ (18)

The solution of this linear fractional differential equation can be obtained using the Laplace transform technique (see also [15])

$$ℒ\left(\frac{d^{\eta }x}{dt^{\eta }}\right)=ℒ\left[\frac{x_{\infty }\left(V\right)-x}{\tau_{x}\left(V\right)}\right]$$ (19)

Where *x*_∞_(*V*) = *α*_*x*_(*V*)/(*α*_*x*_(*V*)+*β*_*x*_(*V*)), *τ*_x_(*V*) = 1/(*α*_*x*_(*V*)+*β*_*x*_(*V*)). Resulting in:

$$s^{\eta }\tilde{X\left(s\right)}-s^{\eta -1}x\left(0\right)=\frac{x_{\infty }\left(V\right)}{\tau_{x}\left(V\right)s}-\frac{\tilde{X\left(s\right)}}{\tau_{x}\left(V\right)}$$ (20)

Where $\tilde{X(s)}$ is the Laplace transform of x and *s* the Laplace space variable. Re-arranging

X(s)˜=x∞(V)τx(V)s(sη+1τx(V))+sη−1x(0)sη+1τx(V) (21)

Using the method of partial fractions and re-arranging:

X(s)˜=x∞(V)s+[x(0)−x∞(V)]sη−1sη+1τx(V) (22)

Note that the inverse Laplace transform of

ℒ−1(sη−1sη−1τx(V))=∑n=0∞znΓ(ηn+1)=Eη(z) (23)

*E*_*η*_ is the Mittag-Leffler function or the generalized exponential function [27]. Therefore, taking the inverse Laplace transform of the entire equation results in [17]

$$x\left(t\right)=x_{\infty }\left(V\right)+\left[x\left(0\right)-x_{\infty }\left(V\right)\right]E_{\eta }\left(-\left[\frac{t^{\eta }}{\tau_{x}\left(V\right)}\right]\right)$$ (24)

### Characterizing power-law behavior in individual gating variables

**Fig 1:**
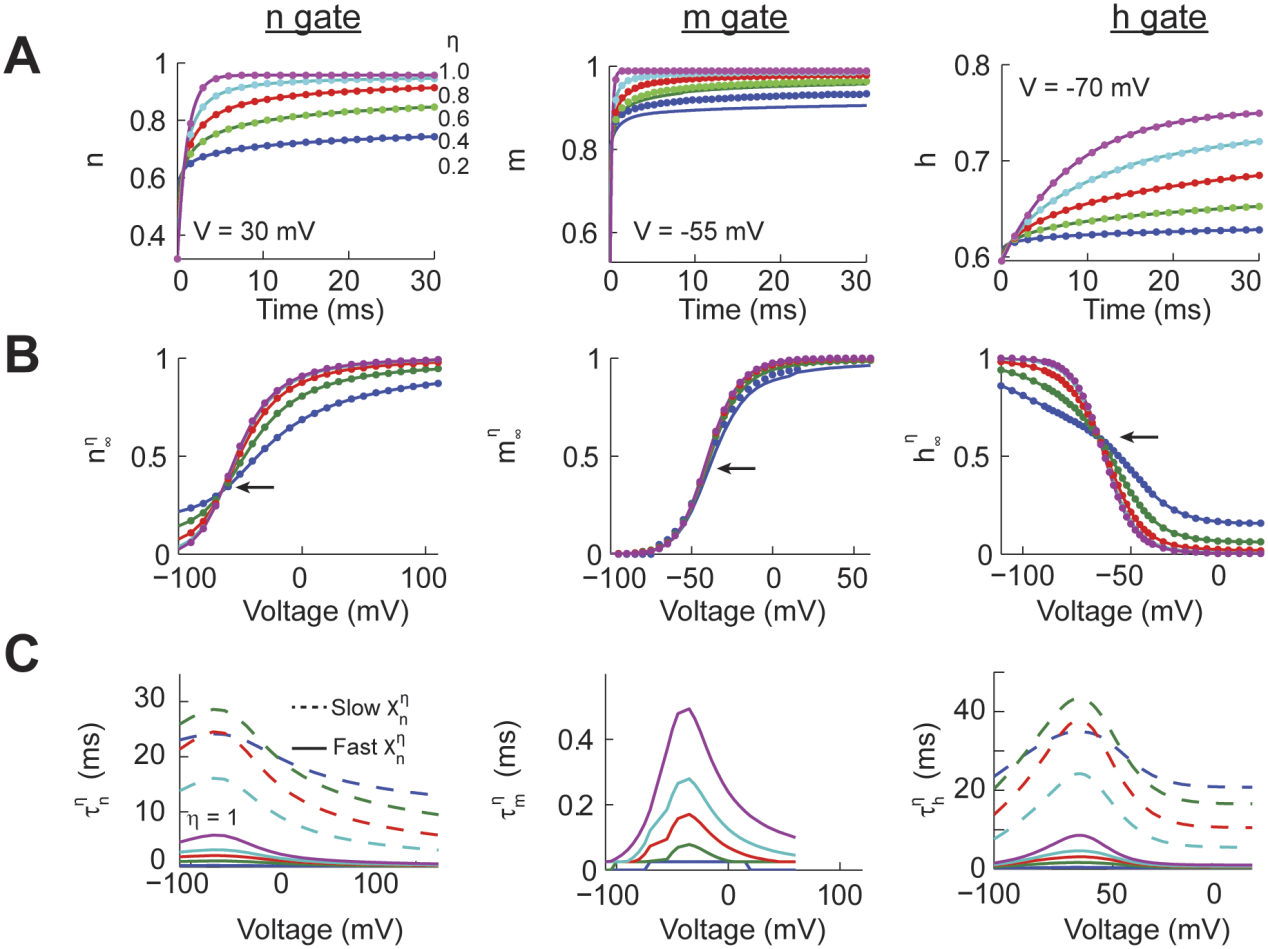
Power-law dynamics of individual Hodgkin-Huxley gating variables. (A) The response of the individual gating variables (n, m, and h) using the analytical (solid) and numerical (dotted) solutions to an identical voltage command (30 mV for n, -55 mV for m, -70 mV for h) with different values of the fraction order derivative (*η*). (B) The long term response of the individual gates ($x_{\infty }^{\eta }$, *x* = *m*,*n or h*) for all the voltages and values of *η* for the analytical and numerical solutions. The arrow points to the inflection point of the sigmoidal curve. For $m_{\infty }^{0.2}$ some numerical solutions were unstable. (C) For the n and h gates we fitted a dual exponential process to the temporal response to voltage commands for all values of *η* resulting in a fast and slow time constant ($\tau_{x}^{\eta }$, x = m, n, or h). We fitted a single time constant to the m gate.

We characterized the response of each one of the power-law behaving activation gates (Eq 18 with x = [n, m, h]) to fixed voltage step commands. The simulations consisted of a period of 20 to 30 ms at a voltage V = 0 followed by the target voltage for up to 100 ms, with target voltages varying from -100 to 120 mV. For a given value of the input voltage command we varied *η* from 0.2 to 1.0. We compared the results of the numerical (Eq 12, Fig 1A dotted line) and analytical (Eq 24, Fig 1A solid line) solutions for all the traces, values of *η*, and voltage commands. The average mean squared error (m.s.e.) between the numerical and analytical solutions for the n gate was 8.2x10^-7^, for the m gate was 2.7x10^-4^, and for the h gate was 9.2x10^-7^. The relatively higher m.s.e. in the m gate traces could be due to the very fast kinetics of this variable which results in deviations from the analytical solution at very short periods of time. In fact, the simulations were unstable for the power-law m gate for values of *η*≤0.2, even when using time steps as small as 10^−5^ ms. In any case, for the large majority of cases our numerical integrations are well matched by the analytical solutions.

In order to quantify the effect of power-law dynamics on each gate we calculated the instantaneous long term response function ($x_{\infty }^{\eta }$, with x = [n, m, h], see definition in explanation of Eq 19). The values of $x_{\infty }^{\eta }$ were obtained from the responses of the respective gates to all combinations of voltage commands and values of *η*. Specifically, to calculate $x_{\infty }^{\eta }$ we measured the value of the power-law behaving gates at t = 90 ms for the n gate, 40 ms for the m gate, and 110 ms for the h gate. These times were chosen because the value of the traces changed by less than 0.01% from the previous millisecond. For *η* = 1 the n, m, and h gates reproduced the classic Hodgkin-Huxley sigmoidal functions (Fig 1B). However, as the value of *η* decreases the slope at the inflection point of the n and h gates become shallower, but not for the m gate (arrows in Fig 1B). Comparing the values of $x_{\infty }^{\eta }$ using our numerical (dotted) and analytical (solid) models shows a very good match. Therefore, power-law dynamics affects the long term response of the n and h gates but has little effect on the fast activating m gate.

A hallmark of a power-law process is that the temporal response of the system cannot be characterized with a single time constant. To illustrate this property we fitted a dual-exponential process to the temporal response of each power-law gate over time windows of up to 100 ms. This fitting process resulted in the calculation of a fast and slow time constant ($\tau_{x}^{\eta }$, x = [n, m, h], see explanation of Eq 19 for a definition). For all the gates when *η* = 1 the $\tau_{x}^{\eta }$ were identical for the fast and slow time constants and to the classic Hodgkin-Huxley model. For the n and h gates as *η* decreases the fast time constant accelerates while the slow time constant slows down, consistent with power-law dynamics. In comparison, the effect of the fractional order derivative on the m gate was fitted with a single exponential process that decreased with lower values of *η*. This suggested that the fast m gate kinetics were only affected over very short periods of time. In summary, power-law dynamics have a strong effect on $\tau_{x}^{\eta }$ and $x_{\infty }^{\eta }$ for the n and h gates, while only having an effect on the fast time constant of the m gate.

### Effects of power-law gating variables on single action potentials

**Fig 2:**
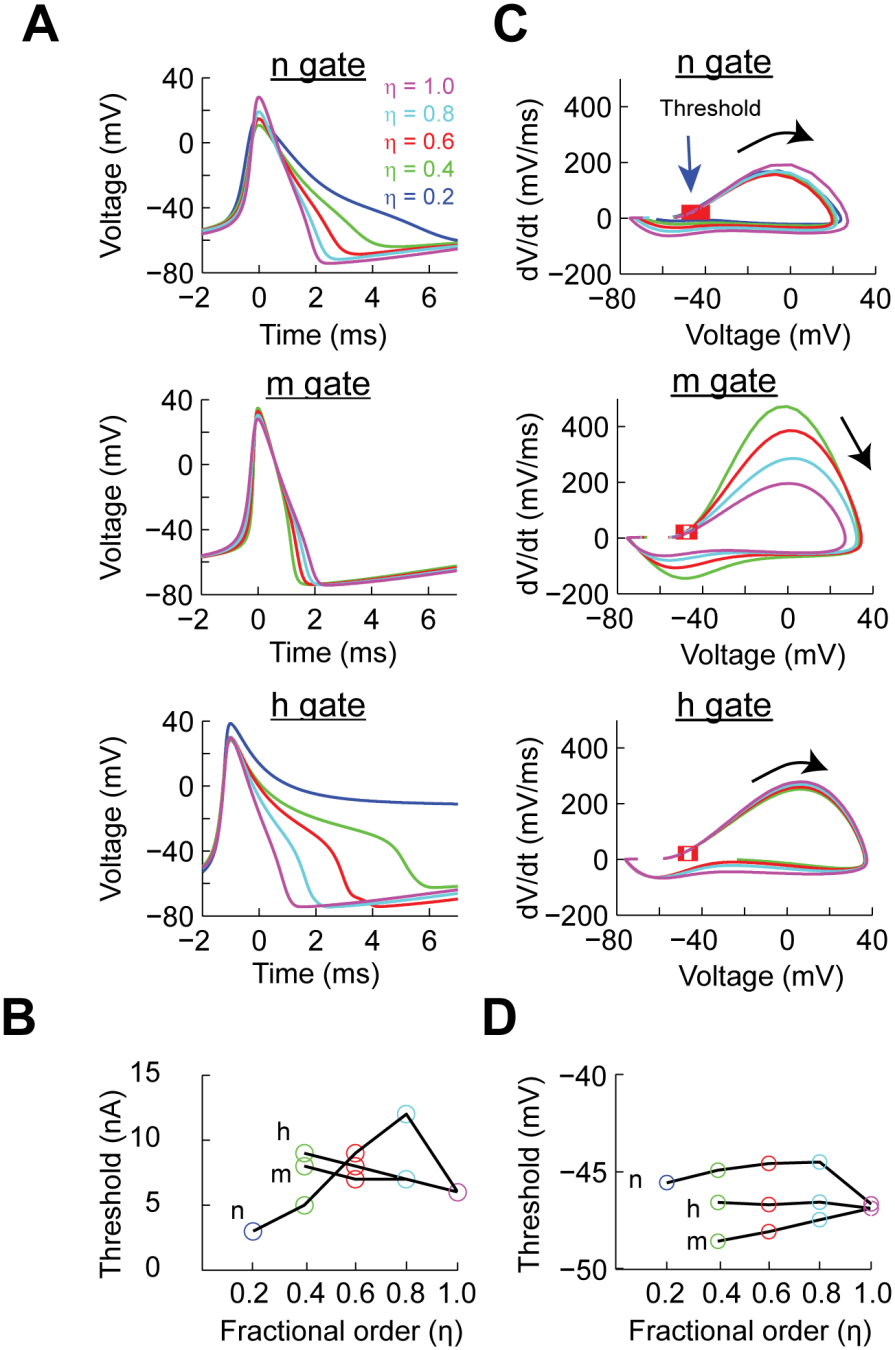
The effect of power-law behaving Hodgkin-Huxley gating variables on the shape and properties of the action potential. (A) Action potential shapes generated with the minimum input current as a function of the order of the fractional derivative (*η*) for the respective gate. (B) The action potential current threshold as a function of input current and *η*. (C) Phase plot of the action potentials generated at minimum input current as function of *η*. The red squares indicate the crossing of threshold detection (dv/dt > 20 mV/ms). (D) The voltage threshold calculated from the phase plots in C.

The shapes of the kinetic curves for each of the gate variables as a function of *η* do not allow to predict whether the complete Hodgkin-Huxley model could produce spikes. In order to test this hypothesis we implemented a full Hodgkin-Huxley model in which a gating variable is governed by fractional dynamics while the other two remained normal. In all simulations we injected a constant current step, from 1 to 24 nA, for 500 ms. We found that action potentials were generated for all values of *η* for each one of the power-law dynamic gates. As is well known, the classical Hodgkin-Huxley model can respond with a single spike before it generates a sustained train of action potentials, with this first shape of the spike being slightly different than the rest [28]. For this reason, we characterized the second generated spike at the minimum input current to elicit spiking for the different values of *η* for each of the activation gates (Fig 2A). In the case of power-law n as *η* decreased the width at half-height of the action potential broadened, from 1.18 ms for *η* = 1.0 to 1.86 ms for *η* = 0.2. There was also a decrease in the minimum value of the repolarization. A similar analysis for the m gate shows that for lower values of *η* the action potential narrows (Fig 2A, m gate). The effect of power-law behavior on the h gate shows a strong effect on the repolarization phase of the action potential (Fig 2A, h gate). As the value of *η* decreases the spike width increases. For *η* = 0.2 the voltage seems to reach a fixed steady state, known as depolarization block. However, as we will show later, this is not the case. Instead, the spiking activity transitions to a pseudo-plateau action potential. Using the same data we calculated the current threshold to generate at least one action potential (Fig 2B). This analysis shows that for power-law dynamics in the n gate the current threshold initially increases and then decreases as a function of decreasing *η*. In contrast, for both power-law dynamic m and h, the current threshold increases. Overall, this analysis shows that fractional order dynamics of the individual gating variables results in the generation of action potentials. Depending on the gate being modified the current threshold of the action potential changes with respect to the classic Hodgkin-Huxley model.

We performed a phase plane study of the action potentials generated at the current threshold. The phase plane analysis is commonly used in experimental work to determine changes in intrinsic excitability [29, 30]. In the case of implementing power-law dynamics in the n gate the overall trajectory of the action potential remains intact with the largest change being the repolarization phase (Fig 2C, n gate). A similar analysis when the m gate has power-law dynamic shows that the speed of the action potential increases as a function of *η* [30, 31] (Fig 2C, m gate). Similar to the n gate, the effect of power-law dynamics on the h gate affects the repolarization phase of the action potentials (Fig 2C, h gate). Phase plane plots are also used in experimental work to determine the voltage threshold by determining the voltage when the speed of the voltage crosses a determined value [30]. In our case we determined the voltage threshold as the value of the voltage when dv/dt>20 mV/ms. This analysis shows that when n has power-law dynamics the voltage threshold increases up to 2.14 mV. In contrast, when the power-law dynamics is in the m gate the threshold decreases by 1.68 mV. As expected from its kinetic properties, power-law dynamics in the h gate has no effect on the voltage threshold (Fig 2D).

The overall analysis of single action potentials shows that spikes can be generated with a wide range of values of *η*. Whenever an action potential is generated the amplitude is similar to the classical Hodgkin-Huxley model. The types of action potentials generated in all cases resemble various types of spikes reported in the literature [29, 32–35], including those from non-neuronal cells [36–38]. Thus, conductances with power-law properties can generate a wide range of action potentials shapes observed in multiple cell types.

### Effects of power-law gating variables on persistent spiking activity

**Fig 3:**
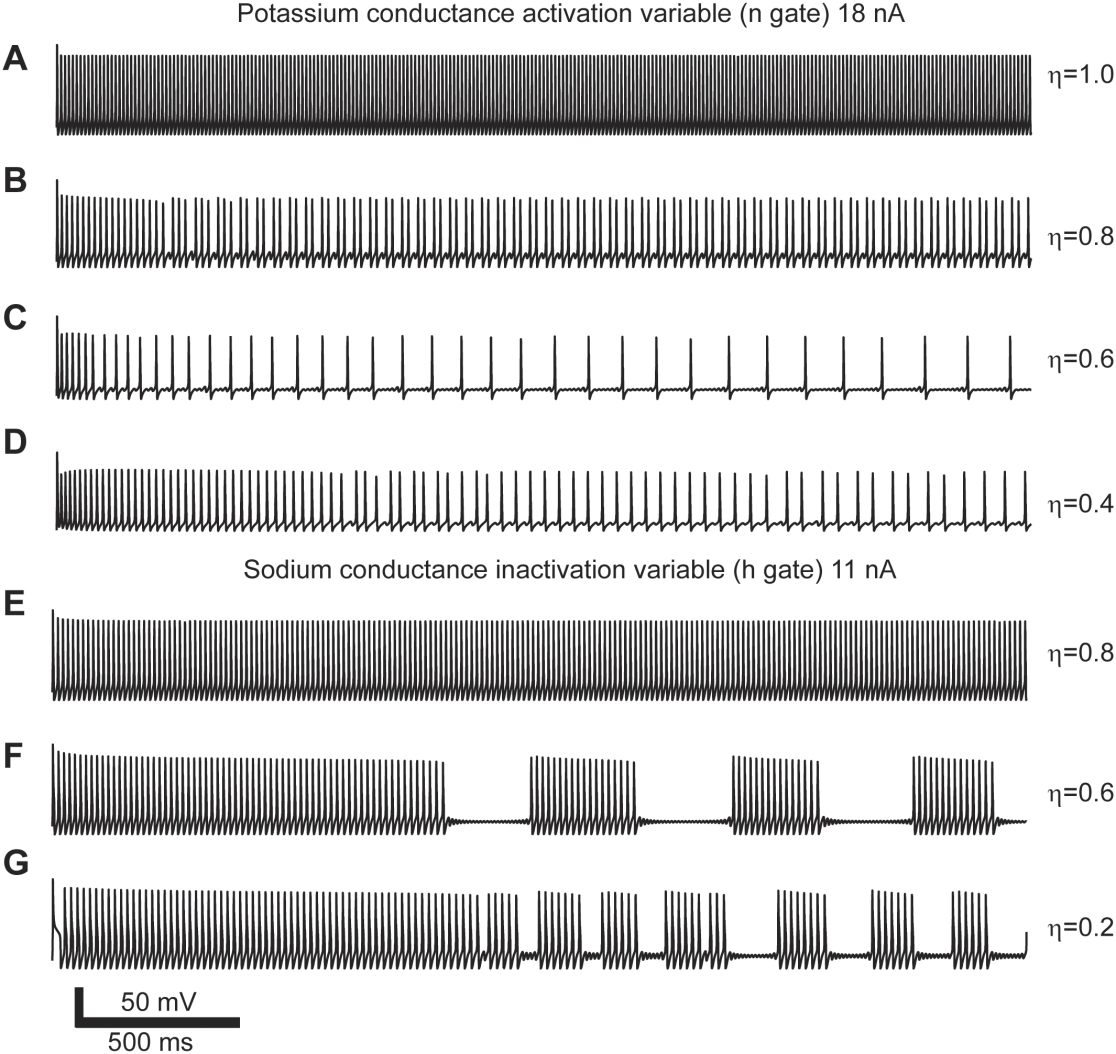
Action potential spiking patterns due to power-law conductances in response to constant current input. (A-D) Spiking patterns generated with power-law behaving n gate. (E-G) Spiking patterns generated with power-law behaving h gate. Each set of simulations done with identical input current and varying the order of the fractional derivative (*η*).

After analyzing the effects of power-law dynamics on individual gates and on the shape of single action potentials we characterized the spiking patterns that emerge from this process. For this purpose we simulated the response of the full model to constant current injection for periods of time between 1,500 to 3,000 ms. For the different combinations of values of *η* and injected current the model showed multiple spiking patterns. For example, for a constant input current of 18 nA we varied the power-law dynamics of the n gate while keeping the m and h gates normal. For *η* = 1.0 the model generated the typical repetitive spiking pattern with a constant firing rate of 84 Hz (Fig 3A). For a value of *η* = 0.8 the number of spikes decreased by almost half and resulted in an average firing rate of 43 Hz. However, the spiking pattern transitioned from repetitive to increasing inter-spike intervals (Fig 3B). For *η* = 0.6 the firing rate dropped to 13 Hz with sub-threshold oscillation between each spike (Fig 3C). Further decrease to *η* = 0.4 also showed sub-threshold oscillations and an increasing inter-spike interval with an average firing rate of 28 Hz (Fig 3D). Another example shows that the effect of power-law dynamics on the h gate also changes the spiking patterns generated by the model in response to constant input. In this case, for a fixed input current of 11 nA and values of *η* ≤ 0.6 the model generates bursts of action potential and sub-threshold oscillations (Fig 3E–3G). These examples show that the power-law behaving conductances results in complex spiking patterns that evolve over time.

**Fig 4:**
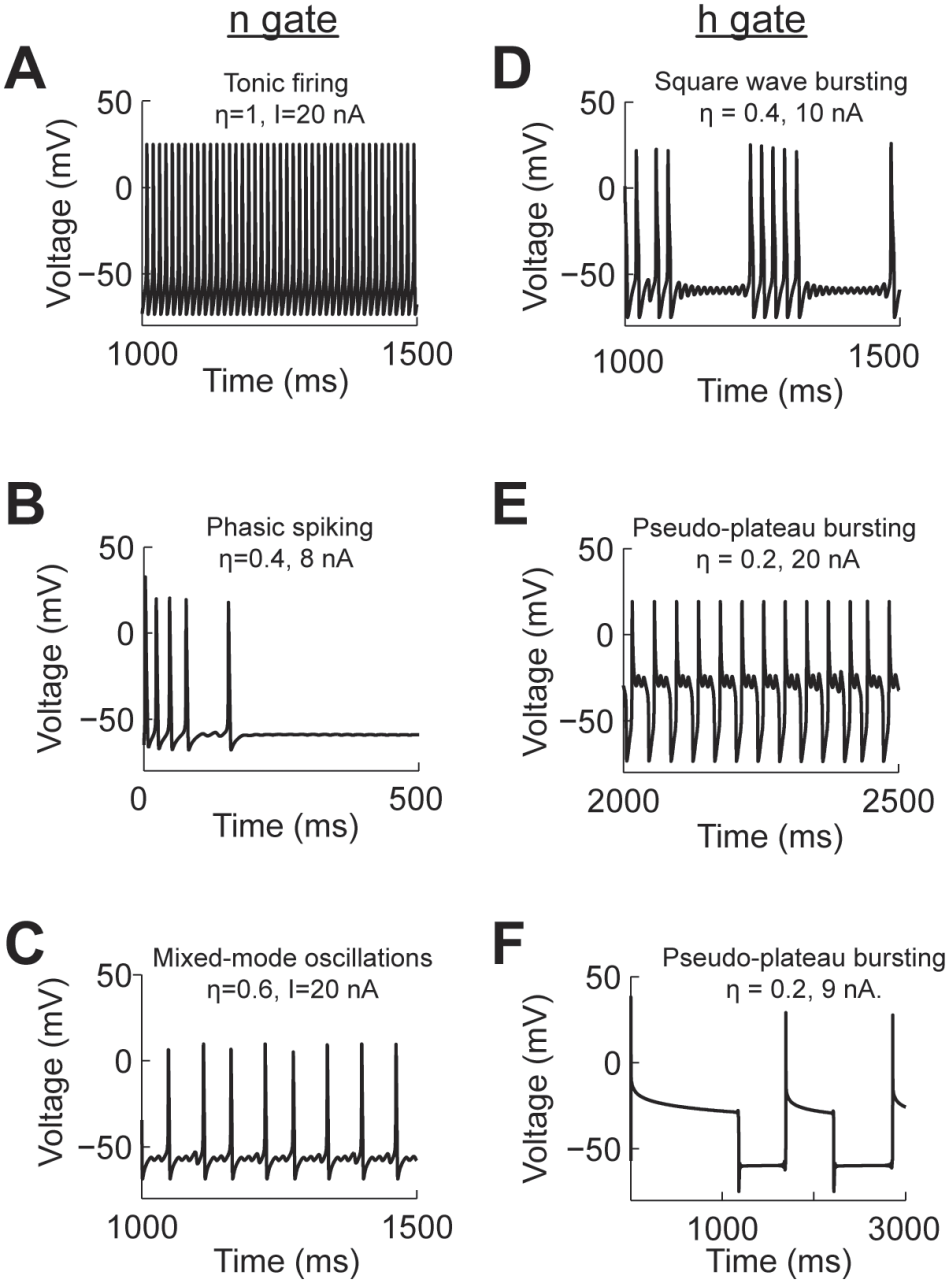
Action potential patterns generated by a Hodgkin-Huxley model modified with power-law behaving n (A-C) and h (D-F) gates. Each panel has the information of the current input (I) and value used for the respective fractional order derivative (*η*).

We classified the spiking patterns generated by the effect of implementing power-law dynamics in individual gates. Since the models could produce non-stationary patterns we decided to classify the spiking activity based on their short (<500 ms) and long term (>1000 ms) responses. We classified the spiking responses as: resting state (RS), no spikes or only one spike at the onset of the stimulus; tonic spiking (TS, Fig 4A); phasic spiking (PS), a few spikes within the first 500 ms (Fig 4B); mixed-mode oscillations (MMO), single spikes surrounded by sub-threshold oscillations (Fig 4C); square-wave bursting (SWB), a group of spikes surrounded by sub-threshold oscillations (Fig 4D); and pseudo-plateau bursting (PPB), long lasting spikes more commonly seen in non-neuronal cells (Fig 4E and 4F) [36–38].

**Fig 5:**
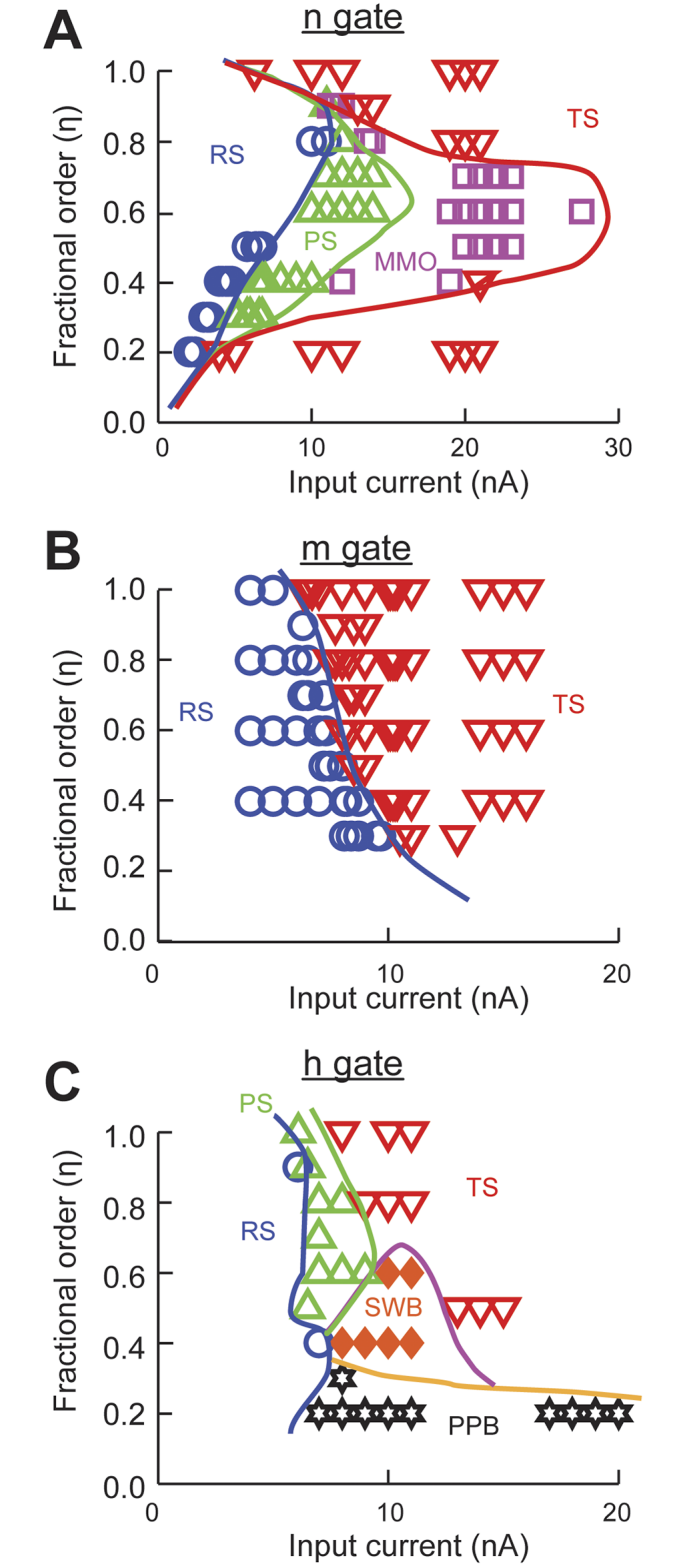
Phase transition diagrams of the spiking patterns generated by the Hodgkin-Huxley model with power-law behaving conductances. The power-law dynamics was implemented with a fractional order derivative of order *η* for the respective gating variables. (A) Potassium conductance activation n gate. (B) Sodium conductance activation m gate. (C) Sodium conductance inactivation h gate. RS, resting state; PS, phasic spiking; MMO, mixed-mode oscillations; TS, tonic spiking; SWB, square-wave bursting; and PPB, pseudo-plateau bursting. Spiking responses and boundaries were manually classified based on the first 1,500 ms of simulation.

We manually classified the spiking patterns generated by the model for a range of input currents from 0–20 nA and *η* = 0.2–1.0. We then produced a spiking pattern phase transition diagram for each of the power-law behaving gating variables (Fig 5). In the case of modeling power-law activation of the potassium channel the phase diagram shows that the spiking activity transitions from RS→ PS → MMO → TS for *η* = 0.3–0.8 (Fig 5A). In all cases, when large input current is applied to the model, this overcomes the dynamics imposed by the fractional derivative and recovers the repetitive firing of the Hodgkin-Huxley model.

The same analysis applied to the activation and inactivation variables of the sodium channel results in very different behaviors. The spiking activity of the model to fractional dynamics of the activation variable, m, results in increased threshold as *η* decreases. After the threshold is crossed tonic spiking results for the duration of the simulation (Fig 5B). When power-law dynamics is applied to the inactivation variable, h, there are multiple spiking patterns that emerge. After the spiking threshold is crossed and for values of *η* <0.8 the system presents SWB and PPB (Fig 5C). For very strong input the neuron spikes regularly (TS) except for values of *η* ≤ 0.2.

In summary, the presence of power-law activation dynamics results in an increase in the diversity of spiking patterns, from tonic spiking to mixed mode oscillations and bursting.

### Attractors due to power-law gate dynamics produce spiking diversity

The numerical solution of the fractional derivative (Eq 12) can be described as a negative feedback mechanism to the value of the gate being computed. The value of the gate at time t is equal to the normal integration of the equation of differences plus a factor that is called the memory trace (Eq 13). When the power-law dynamics of a gate is integrated into the entire Hodgkin-Huxley model then the memory trace acts as a balance between gate activation and action potential generation. To illustrate this point we analyzed the membrane voltage, gate values, and memory traces of several simulations when they generated different spiking patterns.

**Fig 6:**
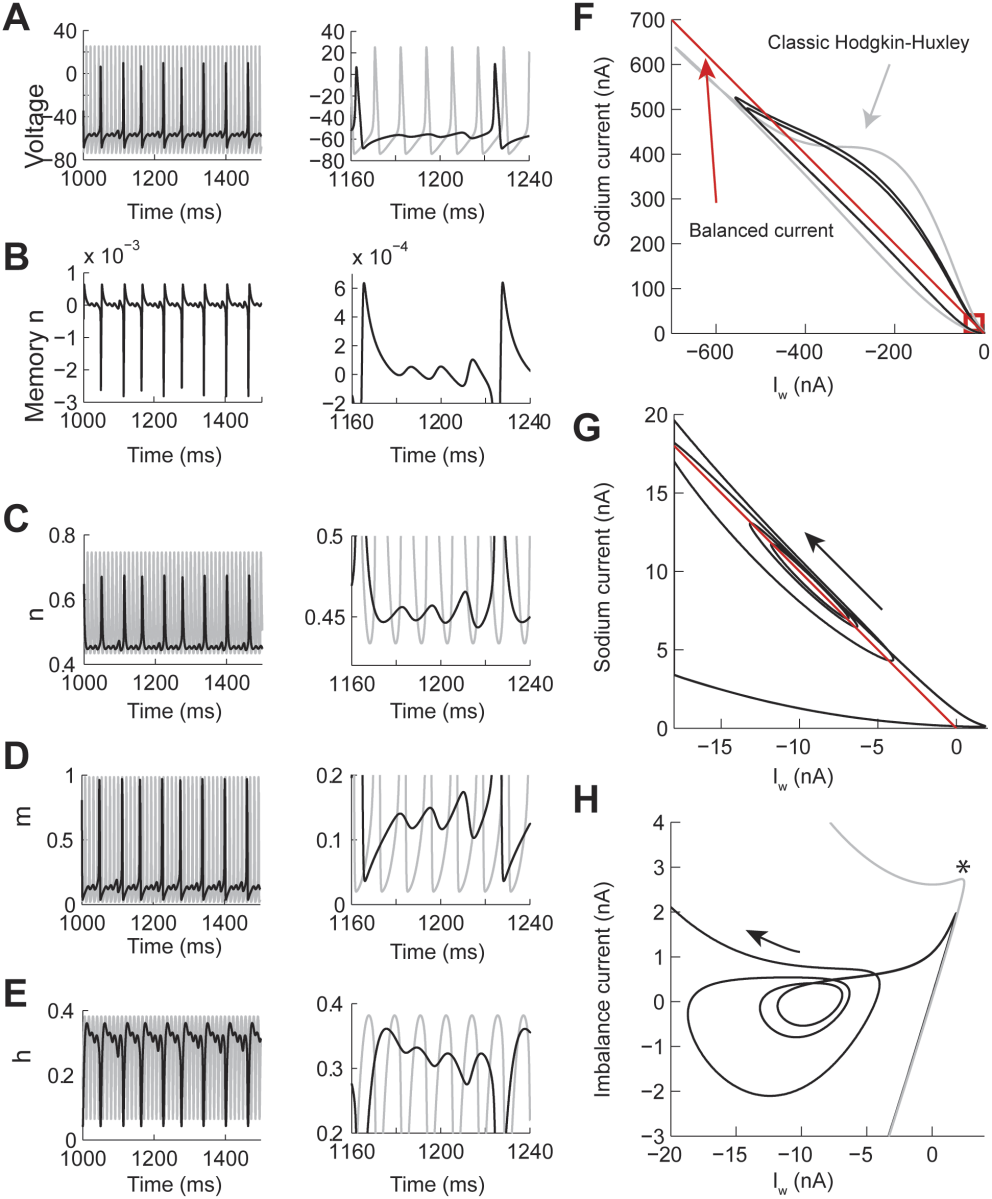
The contribution of the memory trace to Mixed Mode Oscillations in the Hodgkin-Huxley model with power-law n gate. The power-law dynamics was implemented with fractional derivative of order *η* = 0.7 and constant input current I = 23 nA. (A-E) Examples over a long (left) and short (right) time window of the voltage, memory trace, and gate values. The gray line is the identical simulation with *η* = 1.0. (F-H) Phase plane analysis of the same responses. (F) Phase plot of the sodium (I_Na_) vs I_w_ = potassium + leak + injected currents. The red line indicates the balance current and the red square indicates the presence of an attractor. (G) Zoom in the attractor in F. (H) Same data as in G but plotting the imbalance current (I_Na_+I_w_) vs I_w_. The * indicates where I_w_ starts compensating for I_Na_.

As shown before, the MMO patterns are obtained when implementing power-law dynamics in the n gate. We compared the voltage trace of the power-law n, with *η* = 0.7, (Fig 6A, black line) and classic (Fig 6A, gray line) models under the same current input conditions. This shows that the sub-threshold oscillations are not just a process in which the action potential threshold of the classic model is not reached, but that affects the underlying firing rate and spike shape (Fig 6A, right). The memory trace of the n gate shows a negative contribution to the activation of the gate during the action potential depolarization and positive during the repolarization phase (Fig 6B). The negative feedback effect during the generation of the action potential results in a peak value of n smaller than in the classic Hodgkin-Huxley (Fig 6C). As a result the dynamics of the normally activated m and h gates are also modified (Fig 6D and 6E). As shown in Fig 1, the time constant of the potassium conductance decreases over short periods of time. This is due to the positive feedback contribution of the memory trace as the action potential repolarizes. Then this conductance compensates faster for the influx of sodium current, thus blocking the generation of an action potential, instead, producing a sub-threshold oscillation. As the effect of the memory trace vanishes on the n gate then the two currents behave closer to the classical case and an action potential is produced. This dynamics is better understood with a phase plane of the currents involved (Fig 6F–6H). We plotted the value of the sodium current (I_Na_) versus I_w_ = potassium + leak + input currents (Fig 6F, black line). We compared this phase plot to the classic Hodgkin-Huxley model under the same conditions (Fig 6F, gray line). As a reference, we plotted the balanced current between I_Na_ and I_w_ (Fig 6F, red line). Trajectories above this line tend to generate an action potential, while trajectories under this line show that the repolarizing currents are stronger than the I_Na_. At the base of the phase plot we found an attractor that corresponded to the sub-threshold oscillations (red square in Fig 6F and 6G). This attractor has a trajectory around the line of balanced current. To better visualize the attractor we plotted the value of the imbalance current (I_Na_+I_w_) vs I_w_ (Fig 6H). This plot shows that the balance point is around -10 nA. After an action potential is generated then the I_w_ is faster to compensate for I_na_ (* in Fig 6H), bringing the trajectory close to the center of the attractor and oscillate outwards until the potassium conductance returns to a normal state, which then allows the generation of a new action potential.

**Fig 7:**
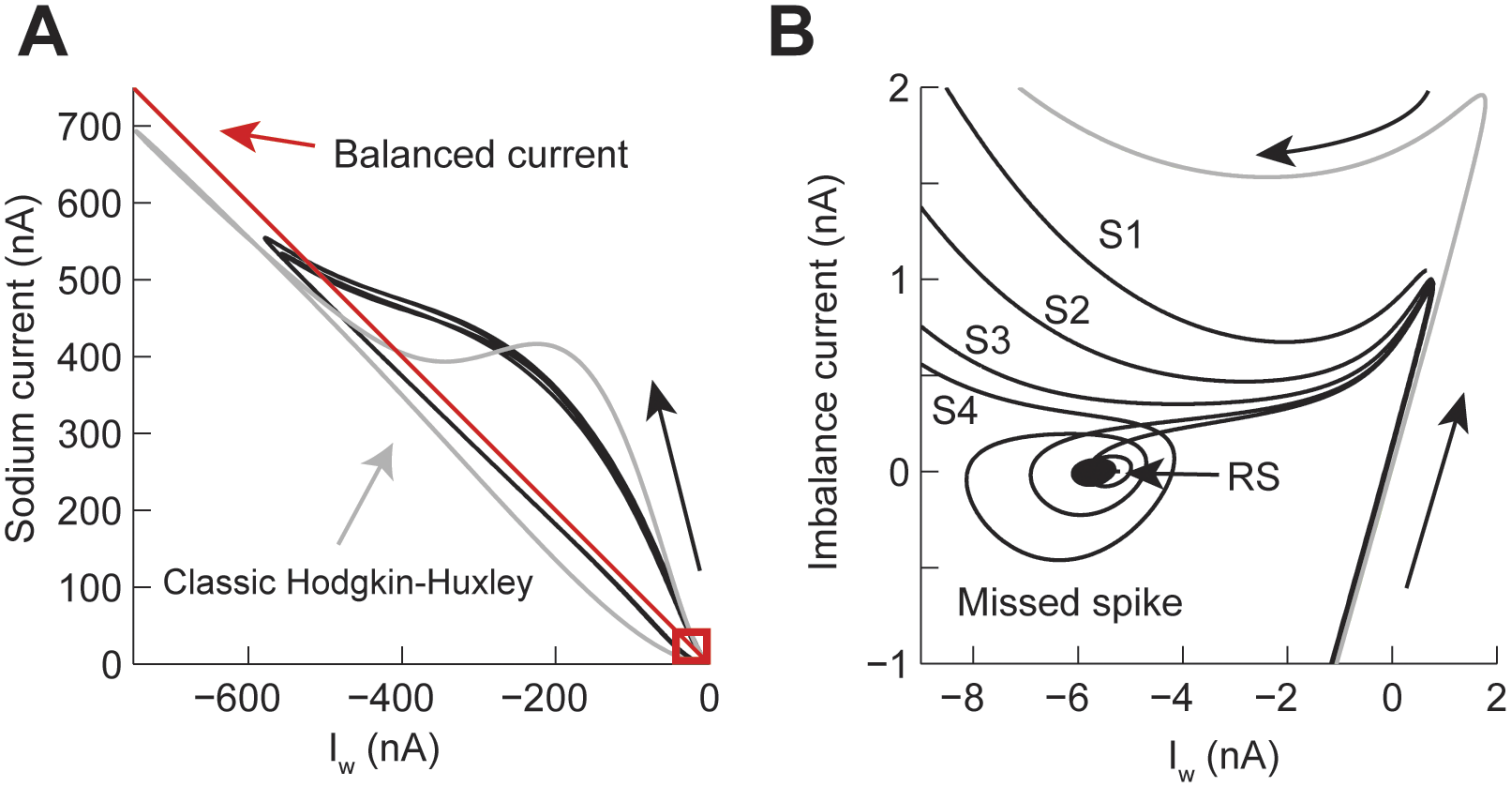
Phase plane analysis of the Transient Spiking pattern (see Fig 4B). (A) Comparison of the current trajectories of the power-law (black) and classic (gray) Hodgkin-Huxley model. The power-law model had a fractional order derivative of *η* = 0.4 and input current I = 8 nA. The red box indicates the area of the attractor. (B) Phase plane of the attractor in A. S1 to S4 indicate spikes and RS is the resting state.

We performed a similar analysis of the PS spiking pattern (Fig 7 with the corresponding voltage trace in Fig 4B). As in the MMO spike pattern the phase plane plot of the I_Na_ vs I_w_ also shows the presence of an attractor at the base of the trajectory (Fig 7A, red square). The current balance point between I_Na_ and I_w_ is close to -6 nA (Fig 7B). As the model generates spikes (S1 to S4 in Fig 7B) the positive imbalance current decreases until the model generates a first sub-threshold oscillation (labeled missed spike in the figure), then a forth spike (S4) is generated, then the trajectory settles into the attractor (RS in the figure). For the duration of this simulation (1,500 ms) no more action potentials were generated; however, it is possible that after the effect of the memory trace on the n gate vanishes the model could start spiking again. The attractors for the MMO and PS patterns are very similar (Figs 6H and 7B). In both cases, the generation of a new action potential is suppressed by a faster compensation of the I_Na_ by the potassium current, which is consistent with an acceleration of the time constant due to power-law dynamics.

**Fig 8:**
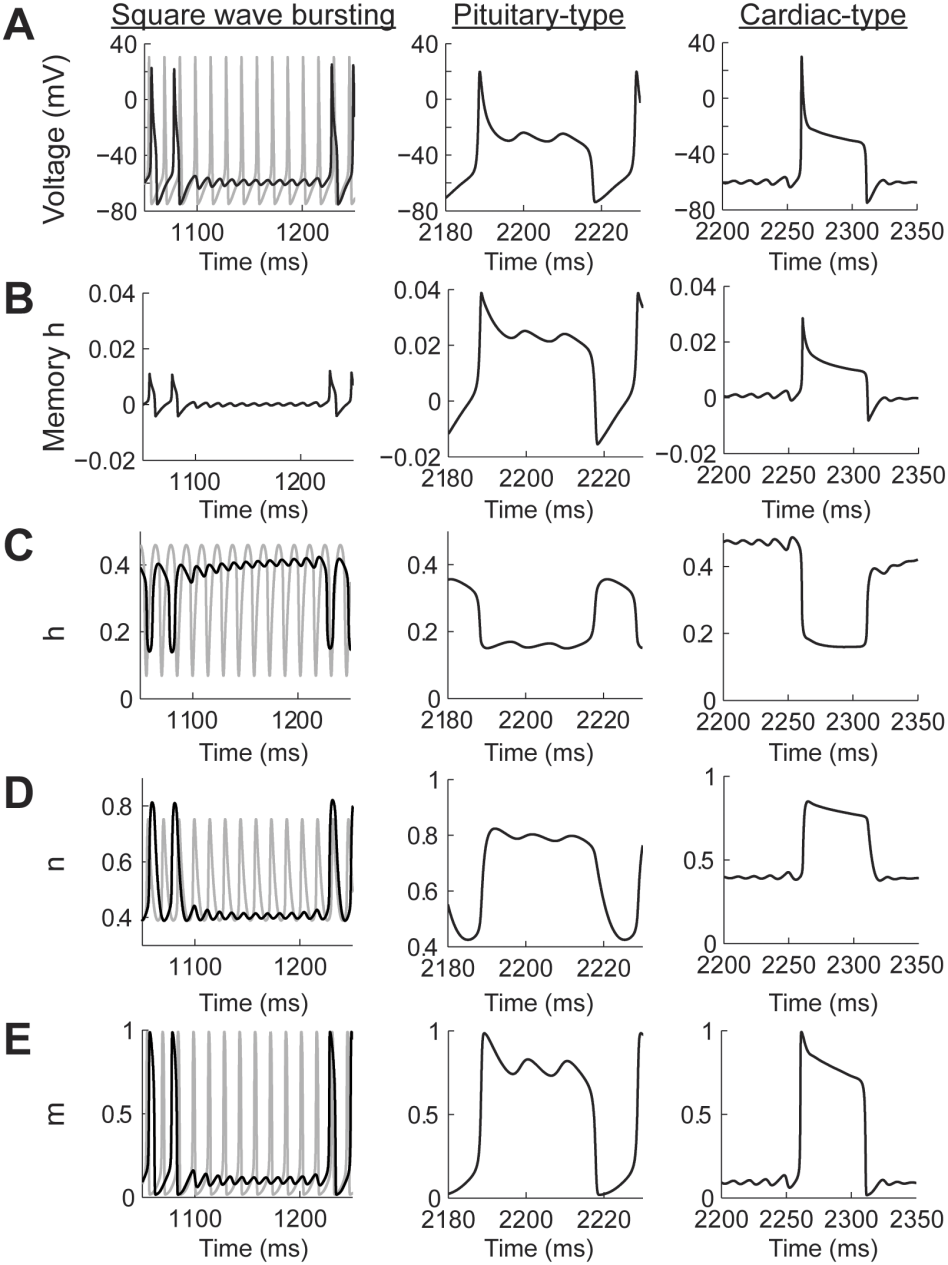
The contribution of the memory trace to square wave bursting and pseudo plateau potential spiking patterns in the Hodgkin-Huxley model with power-law behaving h gate. (A) Voltage traces for square wave bursting and two types of pseudo plateau potential (pituitary and cardiac types). The gray plot corresponds to the classic Hodgkin-Huxley model. (B-E) the temporal behavior of the h memory trace, and gating variables. The square wave bursting was generated with a fractional order derivative of *η* = 0.4 and the input current I = 10 nA; the Pituitary type was generated with *η* = 0.2 and I = 20 nA; and the cardiac type was generated with *η* = 0.2 and I = 9 nA.

Power-law dynamics in the h gate can generate SWB and PPB spiking patterns (Fig 8A) depending on the combination of input currents and values of *η* (see Fig 5C). There are two types of PPB patterns produced by the model. The first one resembles pituitary cell action potentials, which are characterized by a spike followed by high voltage oscillations [36]. The second PPB spiking pattern resembles cardiac myocyte action potentials with a sharp spike followed by a high voltage plateau [37]. Pituitary-type action potentials were generated with higher input currents than cardiac-type action potentials (Fig 8A). In all cases, including the SWB, the amplitude of the memory trace was more than an order of magnitude larger than in the case of the power-law n gate (Fig 8B). In the case of the SWB pattern the spiking activity is slowed down and, as in the case of power-law n gate dynamics, the sub-threshold oscillation do not correspond to just missing spikes from the classic model (Fig 8A, square wave bursting column, black and gay plots, respectively). The effect of the memory trace on the activation of the h gate is to slow down its response when compared to the classic model (Fig 8B and 8C). This slowdown allows the action potential to broaden (cf Fig 2A) and, as a consequence, the maximum value of the n gate is higher than in the classic model (Fig 8D), with the m gate not being affected (Fig 8E). As the effect of the memory trace vanishes from the dynamics of the h gate then the system can again generate a series of action potentials.

In the case of pituitary-type PPB patterns (Fig 8A pituitary-type column) the memory gate also results in a slower activation of the h gate. In this case, this allows the sodium current to remain open for longer periods of time, which compensates for the potassium current, causing an oscillation at a voltage higher than the action potential threshold (Fig 8B–8E Pituitary-type column). As mentioned above, the cardiac-type PPB patterns are generated with lower input currents than the pituitary-type (Fig 8A cardiac-type column). This results in a sharper initial spike and avoids the oscillatory behavior seen for the pituitary-type spiking (Fig 8B–8E cardiac type column). Note that the voltage traces of the pituitary- and cardiac-type spiking patterns show oscillations in different parts of the action penitential. While the pituitary-type has the oscillations in the decaying supra-threshold section of the action potential the cardiac type show sub-threshold oscillations.

**Fig 9:**
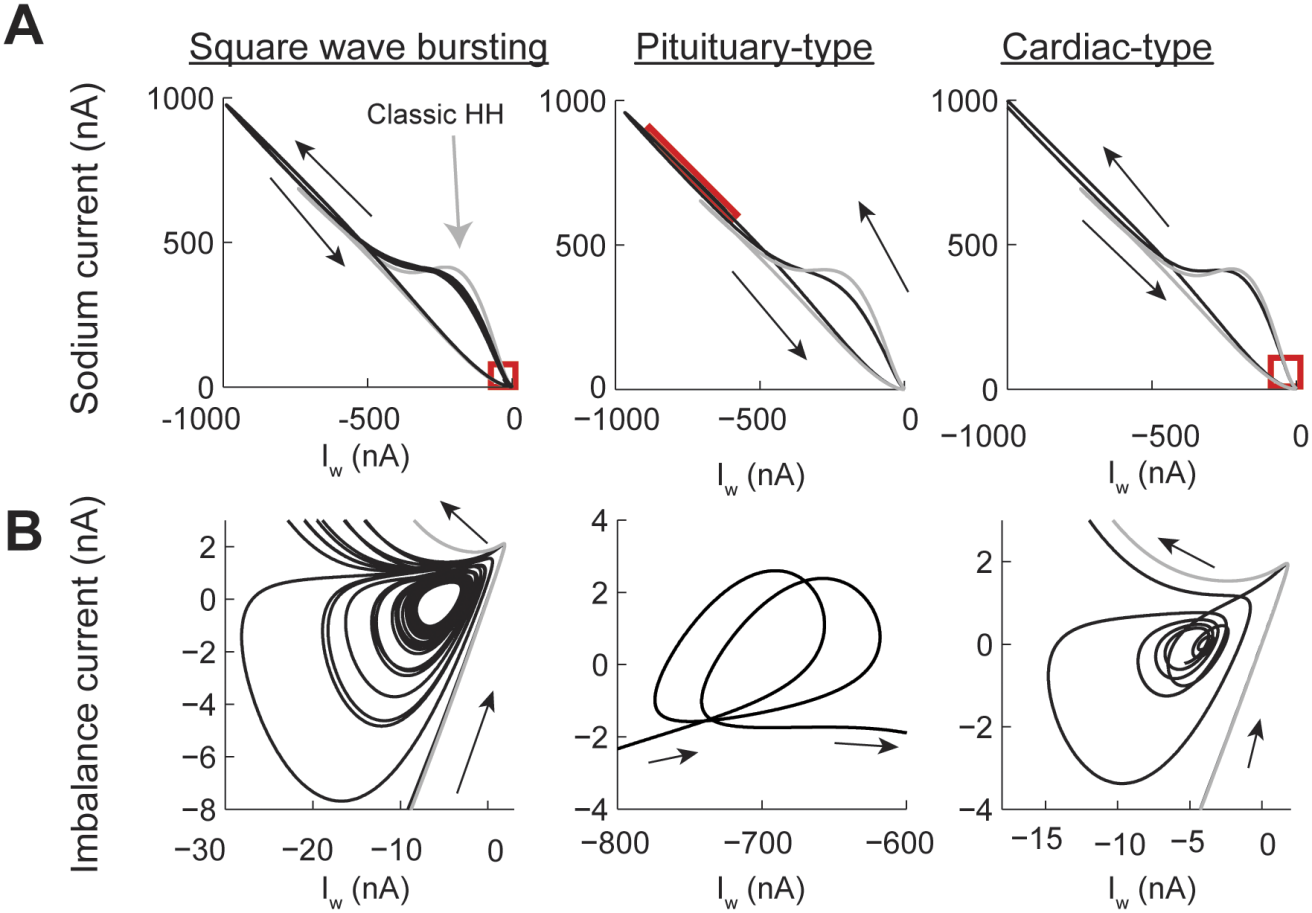
Phase plane plots of the square wave bursting and the two types of pseudo plateau potential spiking patterns in the Hodgkin-Huxley model with power-law behaving h gate. Same settings as in Fig 8. (A) Phase plot of the sodium (I_na_) vs I_w_ = potassium + leak + injected currents, the red square shows the place of the attractor. (B) The attractors from A plotting balanced current (I_na_+I_w_) vs I_w_.

The phase plane analysis of the SWB and PPB spiking patterns confirms that attractors generated by the power-law h gate can appear in different sections of the action potential. In all cases the amplitude of the current generated by the power-law model was larger than in the classic case (Fig 9A). In each one of the trajectories generated we identified the location of the attractors (red boxes). Analyzing the imbalance current phase plane shows that for the SWB pattern the activity is similar to the one of the PS pattern, in which the action potentials during a burst decrease their positive current until the trajectory enters close to the attractor and then spirals out until generating another burst of action potentials. In contrast, in the pituitary-style pattern the attractor is located in the early repolarization of the action potential. Finally, the cardiac-type has a similar trajectory to the SWB and PS patterns, except that the time course of the action potential spreads over a long window of time.

In summary, the effect of the negative feedback of the memory trace on each of the gates variables of the Hodgkin-Huxley model results in the emergence of temporal attractors that balance the depolarizing and repolarizing currents. As a results power-law dynamics of membrane conductances can give rise to a wide range of spiking patterns.

## Discussion

We used fractional order derivatives to study the effects of power-law behaving conductances on the generation of action potentials in the Hodgkin-Huxley model. The fractional order of the derivative provides a memory trace to the past activity of the gate. Our *a priori* hypothesis was that the history dependence on the potassium channel would cause this conductance to have a stronger activation than in the original model and no action potentials would be generated. Similarly, we expected that for power-law behaving sodium activation the model would show depolarization block. However, our systematic computational analysis showed that for a wide range of values of *η*, the model produces spikes with similar amplitude to the classic model. The resulting spike shapes resembled action potentials found in multiple neuronal and non-neuronal cells. The spiking patterns generated in response to constant stimulation also showed an increase in the diversity of responses, such as TS, MMO, SWB, and PPB. Together, our results suggest that power-law behaving conductances can increase the diversity of spike shapes and patterns. We propose that power-law behaving conductances increase the information coding capacity of neurons.

### Power-law conductances and biophysical interpretation

The standard model of a membrane conductance is based on the independence of the open, closed, and inactive states. This assumption is based on a Markov model of protein function. The rate at which a state changes is determined by the voltage and temperature, but not by the previous history of the channel. At the stochastic level this implies that the probability of transition between states depends exclusively on the present state of the system. As a result, the dynamics of the conductance is characterized with an integer order differential equation (*η* = 1).

The Markov model of a voltage or calcium activated conductance is represented by a single open state and multiple closed or inactive states. Power-law activation of such channels can emerge when the number of closed/inactive states is large [5]. In those cases, the state of the channel is assumed to diffuse over the multiple closed states. The time between open episodes depends on the trajectories through the close/inactive states. Under conditions in which the probability of staying in the same state is similar across all states (trapping probability) then the open states follow a power-law distribution. This behavior is equivalent to a random walk with random waiting times, which results in anomalous diffusion, a well-known power-law process [39]. Under this model, each closed/inactive state is still independent and, formally, the process is memory-less. While the transition between states only depends on the present state the emergent behavior is imposed by the complex interactions of a large number of closed states. Thus, the memory trace from the fractional derivative represents the complexity of the distribution in internal states of the channel.

An alternative mechanism to generate a power-law behavior is that there is a small number of internal states that interact with each other. In this case, the transition rates between states not only depend on the present state but on some memory of where the state has been in the past, such as in allosteric processes [40]. At the stochastic level this would mean that the probability of transition changes depending on the previous trajectory of the state. A transition state going from C2→C1→0 with a rate between C1→0 of x would be different if the trajectory were C3→C1→0. The slow power-law activation (*η* < 1) emerges because a state that is closed increases the probability of the next state to remain closed, slowing down the opening of the channels. The memory trace of the fractional derivative represents then how much internal states influence each other, thus deviating from classical Markovian dynamics. Power-law voltage dynamics could also be possible without the sum of multiple membrane conductances but because of actually having fractional order capacitance properties [41]. Thus, a neuron could have independent sources of power-law dynamical properties in the voltage and membrane conductances.

While only using a sodium and potassium conductances our power-law conductance models replicate action potential shapes and activity patterns of multiple cell types. However, some of these patterns are generated by the combination of several conductances. In this context the effect of the power-law dynamics captures the combination of multiple conductances or the different expression of sub-units, which could provide more internal-states or states that interact more strongly.

Our results suggest that it is the potassium or inactivating variables that provide the increase in spiking shape and pattern richness, which is consistent with recent experimental results. For example, different potassium sub-units allow cortical cells to generate firing rate adaptation [13, 42, 43], which we have suggested follows power-law dynamics [14]; the recovery from inactivation of some calcium and sodium channels has been shown to be history dependent [6, 7]; and extended recordings of neurons also show history dependence [8, 44].

### Comparison with other work

In our previous work we implemented power-law dynamics in the membrane voltage of a LIF model. In this model our aim was to replicate the firing rate adaptation reported in multiple types of cortical cells. Instead of increasing the complexity of the model by adding different types of conductances operating in different time domains we proposed that their cumulative effect results in power-law behavior. We showed that with fixed parameters (threshold and membrane resistance) our model replicated a wide array of experimental results by only changing the input current and the value of *η*. Most experiments were replicated with values of *η* < 0.2 [14]. In the present study, the power-law dynamics of the sodium and potassium conductances resulted in changes of the spike shape and spiking patterns that again only depended on the input current and the order of the fractional derivative. The model was consistent with experimental results that suggest that it is the potassium conductances and the recovery from inactivation that allows neurons to generate complex spiking patterns [6, 7, 13, 42, 43]. As such, fractional derivatives can capture the complexity of the combination of multiple conductances or the intrinsic dynamics of individual channels.

A recent study, analyzed the spiking and network properties of a fractional order voltage dynamics Hodgkin-Huxley model [15]. This work showed that applying the fractional order derivative to the voltage reproduces spiking properties not seen in the original model, such as the fast time-to-peak and spike time adaptation. However, this model did not generate complex patterns such as MMO or SWB. This could be due to the effect of the memory trance only on the membrane voltage without affecting the kinetics of the gating variables. In this study it was also found that the range of current inputs that elicit spiking is reduced as a function of the value of decreasing *η*. Although, we find that in our model the threshold to generate spiking varies we found spiking over the entire range of tested values of *η*. Furthermore, whenever action potentials were generated their amplitude was very similar to the classic model.

There are two studies close to our work in which the authors generalized the Hodgkin-Huxley model by applying fractional order dynamics to all the gates [16, 17]. However, these studies were more focused on the application of fractional dynamic analytical and numerical techniques and only analyzed the generation of a single action potential over a narrow range of parameters and values of *η* > 0.65. In contrast, our work systematically studied the response of the model to individual changes of each gate to power-law dynamics over a broad range of input currents and values of *η*. In any case, the numerical techniques used in these and our studies could be incorporated into standard neuronal simulation packages [45].

### Experimental tests to determine if a conductance follows power-law dynamics

The detection of power-law dynamics is a topic of growing interest across the biological sciences [46]. While in stochastic processes detection of a power-law could be complicated by noise, in mesoscopic phenomena, such as in ionic currents in neurons, the measurements can be done more easily; however, experiments have to be designed to be able to detect the existence of power-laws. Isolating single conductances in neurons is experimentally challenging, thus there has to be combination of steps to conclude the existence of power-law behavior:

1. Assuming that a single current can be isolated, perhaps by expressing channels in an oocyte or by pharmacological methods then it is necessary to record for a long period of time (for example 1 sec). Fit a single exponential to the first 1 ms, 10 ms, 100 ms, and 1000 ms. If the fitted time constants are the same then the process is exponential. If the time constant shows a linear relationship with the time window of the fit then the conductance might be following power-law dynamics. The acquisition of the signal should be done at the highest rate possible.
2. Together with the measurement in step A, long term current clamp recordings of action potentials should show the emergence of sub-threshold oscillations. In the majority of cases, this will be MMO that emerge as the neuron adapts. It is necessary to record for long periods of time (longer than 1 sec). These patterns are not going to be static but will continue changing as more spikes are generated.
3. A different way of testing for intrinsic memory in the neuronal spiking generation mechanism is by delivering shorter depolarization steps but at specific intervals. The memory trace for a value of *η* = 0.2 decays 95% in about 800 ms; thus, repeating a series of stimulations separated by 2 sec in the first sweep and by 500 ms in the second would result in the emergence of spiking patterns due to power-law dynamics.
4. If a current is suspected of following power-law dynamics but cannot be isolated, then the conductance could be blocked and use our algorithms together with dynamic current clamp techniques [47] to recover the current with and without power-law properties and compare the results to control experiments.

### Computational consequences

The number of spiking patterns a neuron can generate in relation to its input determines its information capacity [48]. In a Markov process, the spiking activity of a neuron is history dependent as a function of its slowest time constant. This implies that the spiking response, such as firing rate, measures the amplitude or timing of the input. However, if a neuron is constantly integrating inputs and its condition reflects the integration over temporal scales then the spiking activity can vary. Our results show that if conductances follow power-law dynamics then the spiking activity of the neuron will reflect not only the amplitude of the input but how long this input has been delivered, as this would be reflected in the changing spiking pattern. Thus, power-law adaptation increases the computational capacity of neurons. Taking our previous and present results together suggest that power-law dynamics in the voltage or membrane conductances increases the spiking repertoire of a neuron and provides constant adaptation to encode information even in the case of having a small number of conductances.
